# Natural Killer Cells Response to IL-2 Stimulation Is Distinct between Ascites with the Presence or Absence of Malignant Cells in Ovarian Cancer Patients

**DOI:** 10.3390/ijms18050856

**Published:** 2017-05-17

**Authors:** Rodrigo Fernandes da Silva, Adriana Yoshida, Daniela Maira Cardozo, Rodrigo Menezes Jales, Silke Paust, Sophie Derchain, Fernando Guimarães

**Affiliations:** 1 Faculty of Medical Sciences, University of Campinas, 13083-887 Campinas, Brazil; rodrigoinverson@hotmail.com (R.F.d.S.); Adriana122013@gmail.com (A.Y.); Danielamcardozo@gmail.com (D.M.C.); jales@unicamp.br (R.M.J.); derchain@fcm.unicamp.br (S.D.); 2 Center for Human Immunobiology, Department of Pediatrics, Texas Children’s Hospital and Baylor College of Medicine, Houston, TX 77030, USA; silke.paust@bcm.edu; 3 Women´s Hospital “Professor Doutor José Aristodemo Pinotti”–Centro de Atenção Integral à Saúde da Mulher (CAISM), University of Campinas, 13083-881 Campinas, Brazil

**Keywords:** tumor microenvironment, regulatory T-lymphocytes, natural killer activating receptors, CD107a

## Abstract

Peritoneal ascites are a distinguishable feature of patients with advanced epithelial ovarian cancer (EOC). The presence of different lymphocyte subsets has been reported in EOC-associated ascites, which also can or not contain malignant cells. The goal of this study was to analyze the functional characteristics of natural killer (NK) cells from EOC-associated ascites in terms of their expression of activating receptors and ascites’ contents of lymphocyte subtypes, cytokine profile and presence of EOC cells. NK cell function was evaluated by the expression of the degranulation marker CD107a in resting and interleukin (IL)-2 stimulated NK cells from ascites and blood. Degranulation of NK cells from EOC cell-free ascites was significantly (*p* < 0.05) higher than all the other groups, either in their resting state or after IL-2 stimulation, suggesting a previous local stimulation. In contrast, treatment with IL-2 had no effect on NK cells from ascites with EOC cells. The amount of regulatory T cells was significantly higher in ascites with EOC cells compared to EOC cell-free ascites. Ascites with EOC cells also had higher levels of tumor necrosis factor (TNF)-α, suggesting inflammation related to the malignancy. In conclusion, the functional performance of NK cells was distinct between EOC cell-free ascites and ascites with EOC cells. The impairment of NK cell response to IL-2 in ascites with EOC cells was consistent with an immunosuppressive tumor microenvironment.

## 1. Introduction

Epithelial ovarian cancer (EOC) has the highest mortality rate of all gynecological cancers. Its lethality is often due to the advanced stage of the disease at diagnosis. In cases of borderline ovarian tumor or early stage EOC, when the tumor is limited to its location, surgery can benefit 92% of cases. However, 61% of patients present at an advanced stage, and despite therapeutic advances, only 27% survive for 5 years after diagnosis [1]. One of the reasons for late diagnosis is that ovarian cancer is asymptomatic until the occurrence of abdominal bloating or swelling, symptoms commonly associated with ascites and metastases beyond the ovary [2]. Peritoneal ascites are a distinguishable feature of patients with advanced ovarian cancer, and is a result of the inflammatory response triggered by the malignancy. Further, malignant cells are known for their ability to seed the peritoneal cavity and spread through lymphatic vessels that drain the ovaries to the para-aortic and pelvic lymph nodes [3]. Ascites thus contain soluble factors and cells consistent with the tumor microenvironment [4,5,6].

The presence of lymphocytes, either tumor-infiltrating lymphocytes (TILs) or tumor-associated lymphocytes (TALs), in ovarian tumors, has been correlated with disease progression and patient outcome [7]. Specifically, patients treated with surgical resection and chemotherapy, whose tumors contained CD3^+^ T cells, had a significantly higher 5-year survival rate compared to patients whose tumors lacked infiltrating T cells [8,9]. Furthermore, when the subtype CD8^+^ TILs were analysed, they showed a stronger association with prolonged survival than the whole CD3^+^ TIL population [10]. In contrast, high numbers of T regulatory (T-reg) lymphocytes in tumors were associated with shortened survival and impairment of immune functions [11,12,13].

Interestingly, the presence of different T lymphocyte subsets, such as CD4^+^, CD8^+^, and T-regs, has been reported in ascites associated with ovarian cancer, begging the question of whether the immune suppressive tumor microenvironment can and should be extended to the ascites fluid present in these patients. This question is especially important in response to recent reports by Landskron et al. [14], who demonstrated that activated regulatory and memory T cells accumulate in malignant ascites. Thus, the lymphocytes profile in ascites assembles elements from an ongoing immunological response against the tumor, together with the generation of a suppressive microenvironment, but is currently poorly understood. In addition to T-regs, the immune suppressive cytokines interleukin (IL)-10 and transforming growth factor (TGF)-β have also been found responsible for immune suppressive tumor microenvironments [15,16,17,18].

Natural killer (NK) cells are lymphocytes known by their capacity to eliminate neoplastic cells or cells infected by viruses, without previous stimulation [19,20,21,22]. Their cytotoxic activity is determined by a balance of inhibitory and activating stimuli that result from interactions of NK cell-expressed cell surface receptors with their respective ligands on the target cell [23]. Specifically, NK cell-expressed inhibitory receptors recognize HLA class I molecules, and include killer Ig-like receptors (KIRs), CD94/NKG2A, and leukocyte Ig-like receptor B1 (LILR-B1) [24,25]. Activating ligands are recognized by a variety of NK cell-expressed activating receptors, such as DNAX accessory molecule-1 (DNAM-1), natural cytotoxicity receptors (NKp30, NKp44, NKp46), and NKG2D [26,27,28,29]. NK cells also have the capacity to recognize target cells coated with antibodies through antibody-dependent cellular cytotoxicity (ADCC), a mechanism that involves activation of NK cells by Fc-based ligation of the activating receptor CD16 (FcγRIIIa-b) [30].

Although it has been reported that TILs and TALs contain NK cells, little is known about their antitumor functions and their modulation by the tumor microenvironment in ovarian carcinoma, as most of the existing information is derived from NK cells isolated from patient peripheral blood. Initial studies showed that NK cells from patients with ovarian cancer have poor cytotoxic functions against tumors [31,32,33,34], and one study reported that high numbers of infiltrating NK cells in tumors was associated with a worse prognosis [35], which is in conflict with other reports on NK cell infiltration and outcome that focus on different types of cancers [36]. Studies have shown that the antitumor function of NK cells from peripheral blood of patients with ovarian cancer can be significantly augmented by in vitro stimulation with recombinant IL-2 [31,32], and that primary malignant ovarian cells are susceptible to killing by allogeneic NK lymphocytes. However, autologous NK cells do not display significant cytotoxicity in vitro [37,38]. These observations suggest a functional impairment of NK cells in patients who suffer from ovarian cancer, possibly due to the exposure of NK cells to ovarian cancer cells and/or elements present in the tumor microenvironment.

Two studies have recently analyzed NK cells in peritoneal effusions from patients with ovarian carcinoma. In this regard, functional failure of NK cells, was implicated by their defective expression of activating receptors DNAM-1 [39] and NKp30 [40], as a consequence of the overexposure to their ligands, CD155 and B7-H6 respectively. Another mechanism that has recently been implicated in the suppression of NK cells at the tumor site involves their immunomodulation by adenosine. In physiological conditions, adenosine is produced by regulatory T-lymphocytes to modulate immune response [41]. However, the expression of ectonucleotidase CD39 and CD73, and the production of adenosine by breakdown of ATP have been detected in EOC cells [42]. The goal of this study was to characterize NK cells from EOC-associated ascites in relation to their degranulation and response to IL-2. These functional parameters of NK cells were analyzed in terms of their expression of activating receptors and ascites’ contents of lymphocyte subtypes, cytokine profile and presence of EOC cells. For comparison, the same parameters were evaluated in NK cells derived from the blood.

## 2. Results

### 2.1. Degranulation of NK Cells and Their Response to IL-2

**Figure 1:**
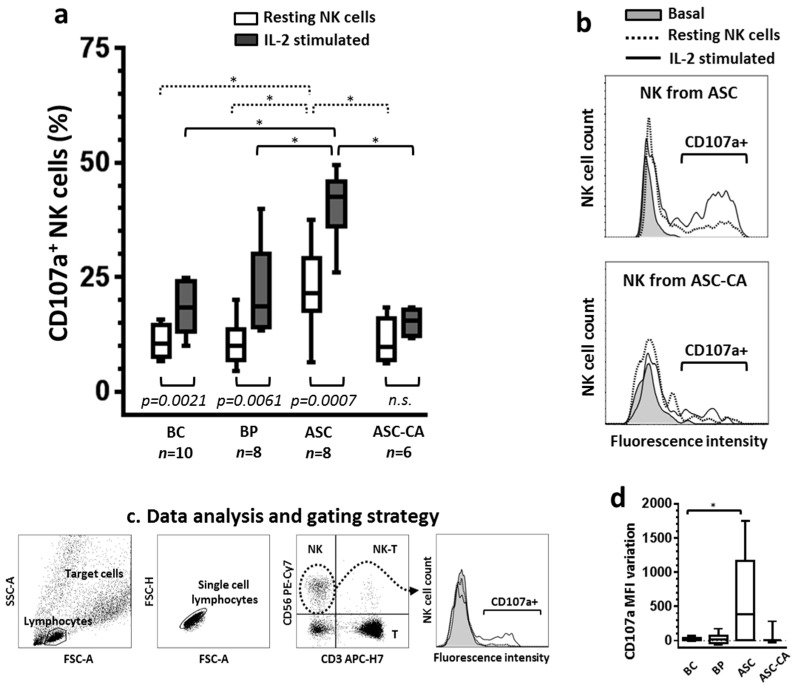
(**a**) Comparison of degranulation between resting and IL-2 stimulated natural killer (NK) cells from blood control (BC), blood of patients with advanced ovarian cancer (BP), epithelial ovarian cancer (EOC) cell-free ascites (ASC) and ascites with EOC cells (ASC-CA). Degranulation was evaluated by the expression of the CD107a molecule on NK cells, resting and after IL-2 stimulation overnight, while coincubated (2 h, ratio 1:1) with K562 target cells. Overnight stimulation with rhIL-2 (1000 UI/mL) was conducted in RPMI-1640 medium supplemented with FBS (10%) and l-glutamine (2 mM). Values are presented in whisker plots as medians; (**b**) Histograms are representative of the CD107a fluorescence intensity profiles of NK cells from ASC and ASC-CA and, the fluorescence intensity levels of the samples were the closest to the mean of the group represented. Basal curve indicates the “background” expression of CD107a of resting NK cells in the absence of target cells K562; (**c**) Flow cytometry-based analysis of NK cell degranulation. To determine CD107a expression, NK cells were gated from the whole lymphocyte population, based on their expression of CD56 molecule and absence of CD3; (**d**) Variation of the mean fluorescence intensity (MFI) was calculated by subtracting CD107a MFI of resting NK cells from CD107a MFI of IL-2 stimulated NK cells. Statistical analyses within groups were performed by Student’s *t*-test for dependent samples; among groups by ANOVA followed by Tukey’s multiple comparisons test, and *p*-values (* *p* < 0.05 on the brackets) indicate significant statistical differences.

The functional characteristics of NK cells from EOC cell-free ascites (ASC), ascites with EOC cells (ASC-CA), blood of control donors (BC) and blood of patients (BP) were evaluated on resting NK cells and after overnight IL-2 stimulation (Figure 1). The percentage of NK cell degranulation, assessed by the expression of CD107a marker, was significantly higher (*p* < 0.05) after stimulation with IL-2 compared to resting NK cells in the ASC, BC and BP groups. In contrast, IL-2 treatment had no significant effect on degranulation of NK cells in the ASC-CA group (Figure 1a), highlighting the inability of ASC-CA-derived NK cells to respond to activating cytokines. Interestingly, degranulation of resting NK cells from the ASC group was significantly higher than resting NK cells of all the other groups, and became even higher after IL-2 stimulation, as indicated by the high percentage of NK cells expressing CD107a (Figure 1a). Additionally, the variation of the mean fluorescence intensity (vMFI) in the ASC group (vMFI = 582.12 ± 682.04) was significantly higher than the BC group (vMFI = 25.98 ± 24.83), but did not differ in relation to the BP group (vMFI = 25.33 ± 82.14) or the ASC-CA group (vMFI = 89.95 ± 167.85) (Figure 1d, vMFI was calculated by subtracting CD107a MFI of resting NK cells from CD107a MFI of IL-2 stimulated NK cells).

### 2.2. Expression of Activating Receptors on NK Cells

**Figure 2:**
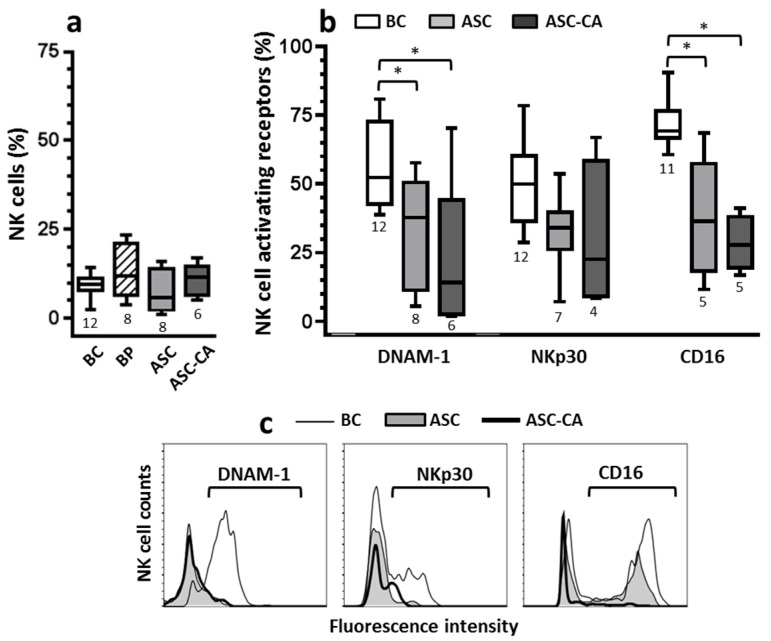
(**a**) Comparison of NK cell frequencies within lymphocytes from blood control (BC), blood from patients with advanced ovarian cancer (BP), EOC cell-free ascites (ASC) and ascites with EOC cells (ASC-CA); (**b**) Comparison of the activating receptors’ expression (DNAM-1, NKp30 and CD16) on NK cells, between ascites (ASC and ASC-CA) and blood from control women (BC). Values are presented in whisker plots as medians; (**c**) Histograms are representative of the activating receptors’ fluorescence intensity on NK cells; the fluorescence intensity levels of the samples were the closest to the mean of the group in each receptor. To determine the activating receptors’ expression, NK cells were gated from the whole lymphocyte population, based on their expression of CD56 molecule and absence of CD3 (see analysis strategy shown in Figure 1c). Statistical analyses for each activating receptor were performed by ANOVA followed by Dunnett´s multiple comparisons test against the control group (BC), and *p*-values (* *p* < 0.05 on the brackets) indicate significant statistical differences.

The frequency of NK cells was evaluated in the BC, ASC, and ASC-CA groups (Figure 2a), as was their expression of the activating receptors DNAM-1, NKp30, and CD16 under the same sampling conditions (Figure 2b). Importantly, the frequency of NK cells expressing activating receptors DNAM-1 and CD16 was significantly reduced in ASC and ASC-CA groups compared to the BC group (Figure 2b). This observation, together with the low fluorescence intensity of DNAM-1, NKp30 and CD16 molecules on NK cells from ASC and ASC-CA groups in relation to the BC group (Figure 2c), indicate down-regulation of important activating receptors, which are known to mediate NK cell antitumor immunity.

### 2.3. Cytokines Profile in Blood Plasma and Ascites Supernatant

**Figure 3:**
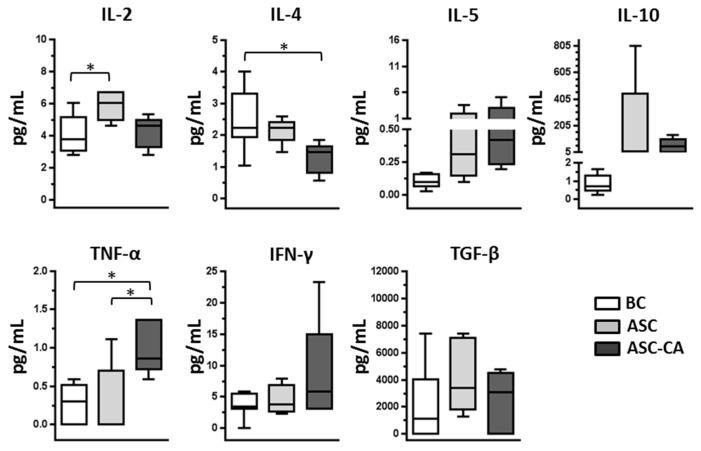
Concentration of cytokines (IL-2, IL-4, IL-5, IL-10, TNF, IFN-γ and TGF-β) in the peripheral blood plasma of controls (BC = 8), ascites supernatant of EOC cell-free ascites (ASC = 5) and ascites with EOC cells (ASC-CA = 5). Values are presented in whisker plots as medians. Statistical analyses were performed by ANOVA followed by Tukey’s multiple comparisons test, and *p*-values (* *p* < 0.05 on the brackets) indicate significant statistical differences.

The concentrations of cytokines IL-2, IL-4, IL-5, IL-10, TNF-α, IFN-γ, and TGF-β were determined in the plasma of the BC group, and the ascites supernatants of the ASC and ASC-CA groups. IL-2 concentration was significantly higher in the ASC group compared to the BC group, and the concentration of IL-4 was significantly lower in the ASC-CA group compared to the BC group. Furthermore, when we investigated TNF-α levels in the ASC-CA group, we found them to be significantly higher in the ASC-CA group compared to all other groups (*p* < 0.05) (Figure 3), possibly as a result of an aberrant inflammatory response to the malignancy.

### 2.4. Phenotype of T Lymphocyte Subpopulations and T-Reg Correlation with NK Cell Function

**Figure 4:**
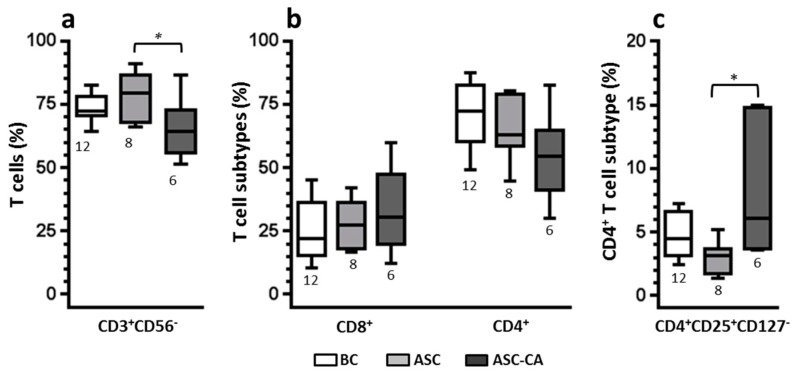
(**a**) Comparison of CD3^+^ T-lymphocyte frequencies among lymphocytes from blood control (BC), EOC cell-free ascites (ASC) and ascites with EOC cells (ASC-CA); (**b**) Comparison of T lymphocyte subsets (CD8^+^ and CD4^+^) within CD3^+^-T lymphocytes; (**c**) Comparison of T-reg lymphocytes subset (CD3^+^CD4^+^CD25^+^CD127^−^) within CD4^+^ T-lymphocytes. Values are presented in whisker plots as medians. Statistical analyses were performed by ANOVA followed by Tukey’s multiple comparisons test, and *p*-values (* *p* < 0.05 on the brackets) indicate significant statistical differences.

**Figure 5:**
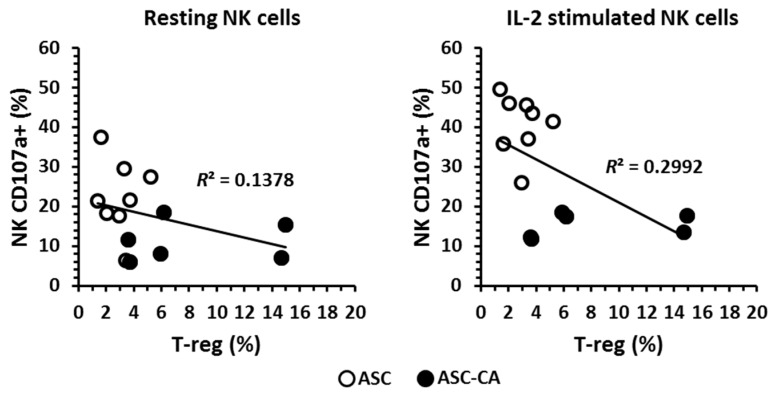
Correlation between T-reg lymphocytes in ascites (ASC, ascites of patients without EOC cells and ASC-CA, ascites of patients with EOC cells) and activated resting or IL-2 stimulated NK cells, represented by the expression of CD107a molecule after co-incubation with K562 malignant cells.

The frequency of CD3^+^ T-lymphocytes (Figure 4a), their subpopulation of T CD8^+^ and CD4^+^ (Figure 4b), and T-reg cells (CD3^+^CD4^+^CD25^+^CD127^−^) (Figure 4c) were determined in the BC, ASC, and ASC-CA groups. A significantly lower percentage (*p* < 0.05) of T cells was observed in the ASC-CA group compared to the ASC group. In parallel, the proportion of T-regs was significantly higher in the ASC-CA group compared to the ASC group. The frequency of T-regs was correlated with the expression of CD107a on resting and IL-2-stimulated NK cells in ascites (Figure 5). A mild inverse correlation was observed in ascites between resting CD107a^+^ NK cells and T-regs (*R*^2^ = 0.1378), and also between IL-2 stimulated CD107a^+^ NK cells and T-regs (*R*^2^ = 0.2992).

## 3. Discussion

The tumor microenvironment in EOC is frequently mentioned as having immunosuppressive properties [18,43]. Cellular and soluble components of the immune system constitute this microenvironment, and their recruitment or production are related to an inflammatory response to the tumor malignancy. In this study, we successfully used ascites from patients with EOC to evaluate the functional characteristics of NK cells and their modulation by the tumor microenvironment. Based on previous studies reporting that NK cells from peripheral blood of patients with ovarian malignancies display poor cytotoxicity [44,45], we hypothesized that NK cells from EOC-associated ascites should also be functionally impaired. However, we observed that ascites can affect NK cells by either promoting or impairing NK function, depending on their contents.

Our results showed that ascites with EOC cells present, as confirmed by cytology results, had a suppressive effect on NK cell function, as measured by the degranulation assay. In contrast, EOC cell-free ascites exhibited the highest level of NK degranulation, either in their resting state or after IL-2 stimulation, suggesting a previous local stimulation. Specifically, this previous stimulation was not only absent in NK cells from ascites with EOC cells, but in addition, these NK cells were hyporesponsive to IL-2 stimulation (Figure 1a, groups ASC and ASC-CA). Additionally, ascites with EOC cells were also characterized by the presence of an elevated percentage of T-reg lymphocytes and inflammatory cytokines, both of which have been associated with the generation of a favorable environment for tumor development in ovarian cancer patients [43,46,47].

The importance of the activating receptors for NK antitumor functions has been demonstrated in patients with ovarian cancer [38,39,40]. As mentioned before, NK cell cytolytic activity is dependent on inhibitory and activating signals resulting from the interaction of their receptors with their respective ligands on target cells. Consistent with Carlsten’s findings [39], our results demonstrated a reduction in NK expression of DNAM-1 and CD16, in EOC-associated ascites. Additionally, and similar to Pesce’s findings [40], our data also indicated a reduction of NKp30 expression in NK cells, although this result has not been statistically significant, possibly due to our limited number of samples. Down-regulation of the NK activating receptors has been explained in terms of their overexposure to their own ligand molecules. Interestingly, the presence of ligands for the activating receptors of NK cells has been demonstrated not only on EOC cells, but also as soluble molecules in ascites and blood of patients [39,40,48]. Studies by Carlsten et al. [36,39] showed that the DNAM-1 ligand, the CD155 molecule, is highly expressed on ovarian carcinoma cells, and overexposure of NK cells to these cells can induce a down-regulation of DNAM-1 by cell-to-cell contact. Similarly, Pesce et al. [40] implicated the B6-H7 ligand molecule with down-modulation of the activating receptor NKp30 in NK cells from ovarian cancer-associated ascites. Thus, both mechanisms have been associated with functional impairment of NK cells in patients with ovarian cancer. Additionally, MUC16 which were found to be elevated in plasma from patients with advanced EOC has been reported to down-regulate the activating receptor CD16 [49]. However, our data showed that NK cells from EOC cell-free ascites (ASC group) had the highest levels of degranulation, either for resting or IL-2 stimulated NK cells, whilst NK cells from ascites with EOC cells (ASC-CA group) were hyporesponsive to IL-2 stimulation. Thus, these findings indicated that in our settings, down-regulation of the activating receptors DNAM-1, NKp30 or CD16, was not, by itself, able to impair NK cell degranulation and their response to IL-2.

With respect to the cytokines, our results showed that cytokine profiles were significantly different between ascites with and without EOC cells, even though concentrations of the cytokines analyzed do not seem to be directly relevant for NK cell function. Although, our cytokine results have been obtained from an accurate evaluation, the levels of cytokines could have been underestimated, due to the ascites generating process. Interestingly, the peritoneum encloses a virtual space, in which its volume can be quickly and dramatically altered during ascites development, mainly in women with advanced ovarian cancer. Such a process might dilute soluble molecules present in the abdominal cavity, and be accounted for the low levels of cytokines observed and their high variability. Our results showed elevated levels of IL-2 in the ASC group compared to the BC group. This is consistent with the high level of degranulation of NK cells from ASC group, and suggests a previous stimulation of NK cells in the tumor site. This observation is also in agreement with our previous study which showed that continuous in vitro stimulation with IL-2 continuously increases NK activation [38]. Elevated TNF-α levels observed in ascites with EOC cells suggests an inflammatory process that possibly benefits tumor development. The contribution of inflammatory cytokines to tumor development has been investigated by other studies [43,47,50]. In particular, in ovarian cancer, Kulbe et al. [46] showed that in mice, TNF-α secretion by EOC cells stimulates a constitutive network consisting of cytokines (including IL-6 showed in Figure S1), chemokines, and angiogenic factors, which promotes colonization of the peritoneum and neovascularization for the development of tumor implants. In our advanced-stage EOC patients, a similar process might be occurring in the tumor microenvironment.

Moreover, the presence of high numbers of T-regs in ascites with EOC cells corroborates the idea that there was an ongoing inflammatory process. T-regs are known to counteract inflammation by controlling immune cells and enabling maintenance of tissue homeostasis [41]. Similar to our results, Landskron et al. [14] showed the accumulation of T-regs in ascites from EOC patients, which positively correlated with the contents of EpCAM^+^ cells in ascites. They also confirmed proliferation and recruitment of T-regs towards ascites by the expression of CCR4 and Ki-67, and activation by the expression of CD147 and CTLA4 [14]. The T-regs observed in our ascites samples were CD4^+^CD25^+^CD127^−^ cells, which is a phenotype consistent with activated T-regs, since in T-regs the CD127 molecule inversely correlates with FoxP3, a well-known marker for activation of immune cells [51,52,53,54].

T-regs have the ability to secrete IL-10 and TGF-β to modulate helper and cytotoxic T-lymphocytes, dendritic cells, monocytes, and B lymphocytes [55,56,57], as well as through cell-to-cell contact by the interaction of CTLA on T-regs with CD80 and CD86 on dendritic cells [58]. Ghiringhelli et al. [12] showed in an in vitro system that the cytolytic activity of NK cells was impaired by soluble TGF-β or T-reg lymphocytes, and by using anti-TGF-β as a blocker, NK cell cytolytic activity was restored. Similarly, Smyth et al. [13] also showed that anti-TGF-β could restore NK cell cytolytic activity in the presence of T-regs. However, they indicated that cell-to-cell contact, i.e., mediated by TGF-β on the T-reg surface, was necessary, since co-cultivation of T-reg and NK cells in a transwell system, or the supernatant of activated T-regs, had no inhibitory effect on NK cell activity. However, a recent study by Viel et al. [59] provided evidence that TGF-β inhibits the activation of NK cells by repressing the mTOR pathway. Interestingly, they showed that suppression of TGF-β signaling in NK cells enhanced the ability of NK cells to control metastases in murine models.

In our study, ascites with EOC cells were the only group with impaired NK cell response to IL-2, as demonstrated by the degranulation assay. Considering that the levels of TGF-β and IL-10 were similar between EOC cell-free ascites and ascites with EOC cells, and the amount of T-regs was significantly higher in ascites with EOC cells, the presence of T-regs is most likely implicated in the impairment of NK cell response to IL-2 than the presence of soluble forms of TGF-β and IL-10. Moreover, our data on the correlation between the amounts of T-regs in ascites and NK cell degranulation (Figure 5) indicated that, in our system, T-regs might exert a mild modulatory effect on NK cell degranulation, around 30% of the whole effect (or *R*^2^ = 02992), in NK cells that were stimulated with IL-2. Instead, when EOC cells were present in ascites, stimulation with IL-2 was ineffective to increase NK cell degranulation.

Our data provide insights into the paracentesis procedure to remove ascites fluid, often conducted in patients with advanced ovarian cancer. EOC cell-free ascites with functional NK cells could be beneficial to the patient, however, ascites containing EOC cells with impaired NK cell function, and correlated with an increase in T-regs, most likely negatively affect the local immune response to the tumor. Therefore, the removal of ascites should be carefully considered, since it could affect an ongoing beneficial immunological response in the peritoneal cavity of EOC patients. In conclusion, the functional performance of NK cells was distinct between EOC cell-free ascites and ascites with EOC cells. In contrast to EOC cell-free ascites, ascites with EOC cells displayed an immunosuppressive tumor microenvironment, with high contents of T-reg lymphocytes, down-regulation of NK cell-activating receptors and NK hyporesponsiveness to IL-2 stimulation, as demonstrated by the degranulation assay. Among the suppressive mediators in ovarian cancer-associated ascites, the presence of EOC cells seems to play a role in the impairment of NK cells’ response to IL-2 stimulation. However, whether EOC cells in ascites are the cause or consequence of NK cells’ hyporesponsiveness remains to be clarified in future studies.

## 4. Materials and Methods

### 4.1. Patients and Samples

For this study, we included 14 EOC patients with ascites, treated in the Pelvic Oncology Clinic and scheduled for surgical intervention, and 12 healthy women (controls) matched in age (57.8 ± 11.6 years and 60.2 ± 12.9 years, respectively), treated in the Menopause Clinic of the Women’s Hospital of the University of Campinas (Campinas, Brazil). Among the 14 ascites samples, six ascites displayed malignant ovarian cells through cytology evaluation. Additionally, serum biomarkers such as MUC16 are an important component in the workup of women with adnexal masses. In the Women’s Hospital of the University of Campinas, MUC16 is routinely evaluated in blood of women assisted in Pelvic Oncology Clinic. As expected, the levels of MUC16 were elevated in serum from patients with EOC associate ascites which were included in the study (CA125 = 1535.5 ± 1250.6 U/mL, ranging from 126 to 3963 U/mL). However, MUC16 was not assessed in ascites. The study was approved by the Research Ethics Committee of University of Campinas (27 September 2011, #897/2011) and was registered on the Brazilian National Health Council (CAAE: 0807.0.146.000-11). Signed informed consent was obtained from all patients. Specific patient characteristics at the time of sample collection are provided in Table 1.

Blood samples were collected using 10 mL vacuum blood-sampling tubes containing sodium heparin (Vacuette, Campinas, Brazil). Ascites samples from patients with ovarian cancer were collected by ultrasonography-guided paracentesis, quickly transferred to 50 mL conical tubes, and sodium heparin added (5 µL/mL of heparin; liquemine 5000 UI/mL, Roche, Rio de Janeiro, Brazil) under sterile conditions. Ascites samples were classified as ascites without EOC cells, or ascites with EOC cells. Initially, 1 mL of every sample of blood and 5 mL of every sample of ascites were transferred to new conical tubes and centrifuged at 600× *g* for 5 min to obtain cell-free plasma and ascites fluid, respectively. Then, the resulting supernatants were kept frozen (−20 °C) until used for cytokine quantification.

Subsequently, peripheral blood mononuclear cells (PBMC) and the cellular fraction of the ascites were isolated by gradient centrifugation, using Ficoll-Paque Plus (GE Healthcare, Uppsala, Sweden), followed by a washing procedure performed twice (centrifuged at 600× *g* for 5 min) using a balanced salt solution (DMPBS-FLUSH; Nutricell Nutrientes Celulares Ltda, Campinas, Brazil). Cell numbers were assessed in a Neubauer chamber using acetic acid solution (2% *v*/*v* in PBS) and the trypan blue (1% *w*/*v* in PBS) exclusion method to assess viability. Replicates of the resulting cell pellets were cryopreserved in fetal bovine serum (FBS; Nutricell Nutrientes Celulares Ltda) containing 10% DMSO (Sigma, St. Louis, MO, USA), for subsequent phenotyping of the lymphocytes and evaluation of NK cell function.

Thus, four experimental groups were defined based on the characteristics of the samples: Ascites of EOC patients without malignant cells also mentioned as EOC cell-free ascites (ASC, *n* = 8); ascites of EOC patients with malignant cells (ASC-CA, *n* = 6); blood of control donors (BC, *n* = 12); and blood of patients (BP, *n* = 8). Given to the management strategy for blood sampling, the BP group had blood from eight out of the 14 patients from whom ascites were obtained (four patients of the group ASC and four patients of the group ASC-CA).

### 4.2. Resting and Overnight rhIL-2 Stimulated Effector Cells

Cryovials with ascites cells (ASC and ASC-CA groups) or PBMC (BC or BP groups) were removed from the liquid nitrogen, thawed at room temperature and washed twice with PBS. After washing, cell number and viability were assessed and the cell suspension was adjusted to a density of 1 × 10^6^ cells/mL with RPMI-1640 (Nutricell Nutrientes Celulares Ltda) supplemented with 10% FBS and 2 mM l-Glutamine. Each of the cell suspensions obtained by this procedure was split into two equal parts, and 1000 U/mL of recombinant human IL-2 (rhIL-2) was added to one of them. Both samples were then incubated overnight at 37 °C in 5% CO_2_.

### 4.3. K562 Cell Line Target Cells

The K562 (human erythromyeloblastoid) cell line, originally obtained from the American Type Culture Collection (ATCC, Rockville, MD, USA), is routinely maintained in the laboratory and phenotyped for its usual surface markers, particularly CD45^+^ and HLA^−^. The K562 cells are cultured in vitro in RPMI-1640 medium supplemented with 10% FBS, 2 mM l-glutamine, and replenished with fresh medium every 2–3 days.

### 4.4. NK Cell Degranulation Assay and Activating Receptor Phenotyping

The functional characteristics of NK cells were evaluated by a standard flow cytometric degranulation assay, which is based on the quantification of cell surface-expressed CD107a (LAMP-1) for the visualization and quantification of activated NK effector cells present in blood and ascites. This method has been described previously by Bryceson et al. [60], for the evaluation of NK cell functionality during target cell lysis. Effector cells (resting and IL-2-stimulated cells) and target cell suspensions (K562 cell line) were prepared at a concentration of 2 × 10^6^ cells/mL. The effector cell suspensions were coincubated with target cells in a 1:1 ratio and in a final volume of 200 µL, in U bottom microtubes (Jetbiofil, Guangzhou, China), in duplicate. Cells were spun down quickly (30× *g* for 3 min) and incubated for 2 h at 37 °C. Tubes containing effector cells without target cells were also prepared for quantification of basal expression of CD107a. After incubation, microtubes were centrifuged (600× *g* for 5 min), supernatants discarded and cell pellets suspended in 50 µL of staining solution (PBS supplemented with 2% FBS and 2 mM EDTA) containing the fluorochrome-conjugated monoclonal antibodies (mAb) anti-CD3 APC-Cy7 (clone SK7), anti-CD56 PE-Cy7 (clone B159), anti-CD107a PE-Cy5 (clone H4A3), anti-DNAM-1 FITC (clone DX11), anti-NKG2D APC (clone 1D11) (BD Pharmingen™, San Jose, CA, USA), and anti-NKp30 PE (clone AF29-4D12) (Miltenyi Biotec, Bergisch Gladbach, Germany). Cells were incubated for 30 min on ice and protected from light. Cells were then washed twice (centrifuged at 600× *g* for 5 min), re-suspended in 400 µL PBS and subjected to flow cytometry analysis. Data acquisition was performed using a FACSVerse cytometer with FACSuite software (Becton Dickinson, San Jose, CA, USA). Between 10,000 and 20,000 cells were acquired. Data analysis was conducted using FlowJo software (Version 10), Tree Star, Ashland, OR, USA). The lymphocyte population was identified by the forward scatter (FSC) and side scatter (SSC) parameters, then FSC-Area vs. FSC-Height was used to eliminate doublets. Within the lymphocyte population, NK cells were identified by anti-CD3 APC-Cy7 vs. anti-CD56 PE-Cy7 and gated on CD3-negative CD56-positive cells. Within the NK cell population, the parameter anti-CD107a PE-Cy5 was analyzed to quantify degranulation levels (Figure 1c). A similar gating strategy was used for the quantification of activating receptors within the NK cell population.

### 4.5. Lymphocyte Phenotyping

Lymphocytes present in PBMC (BC group) and ascites (ASC and ASC-CA groups) were phenotyped for the identification of their subsets. A flow cytometric-based assay was used according to standard procedures [37]. Briefly, the cells were mixed with 50 µL of staining solution containing a mix of fluorochrome-conjugated monoclonal antibodies at a 1:50 dilution; anti-CD3 APC-Cy7 (clone SK7), anti-CD4 PerCP-Cy5.5 (clone RPA-T4), anti-CD25 PE (clone M-A251), anti-CD56 PE-Cy7 (clone B159), anti-CD127 Alexa Fluor647 (clone HIL-7R-M21) (BD Pharmingen™), and anti-CD8 FITC (clone OKT8) (Miltenyi Biotec). Cells were incubated for 30 min on ice and protected from light. After the incubation, cells were washed twice with PBS and the final pellets suspended for acquisition in a FACSVerse cytometer using the FACSuite software (Becton Dickinson, San Jose, CA, USA). FlowJo software was used for the data analysis. The lymphocyte population was identified by the FSC and SSC parameters, and then FSC-Area vs. FSC-Height was used to eliminate doublets. Within the lymphocyte populations, the CD3^+^ lymphocyte population was identified by anti-CD3 APC-Cy7. Within the CD3^+^ lymphocytes, CD4^+^ and CD8^+^ populations were distinguished. Within the CD4^+^ population, the T-reg population was quantified by the parameters anti-CD25 PE vs. anti-CD127 Alexa Fluor647.

### 4.6. Cytokines Profile

The presence of cytokines in blood plasma (BC group) and ascites supernatant (ASC and ASC-CA groups) was determined by the CBA assay (Cytometric Bead Array, BD Biosciences, San Jose, CA, USA). The kits used for the analysis of cytokines were the Th1/Th2 CBA kit, specific for human IL-2, IL-4, IL-5, IL-10, TNF-α, and IFN-γ; and the Single Plex Flex Set CBA kit, specific for human TGF-β1. Both experiments were conducted according to BD Biosciences manufacturer’s protocol. Briefly, appropriate volumes of unknown samples (plasma or ascites fluid) were simultaneously incubated with capture bead conjugated with a cytokine-specific antibody and the detection reagent (phycoerythrin(PE)-conjugated antibody). As a result, sandwich complexes (capture bead + cytokine + detection reagent) are formed, which can be measured using flow cytometry. The intensity of PE fluorescence of each sandwich complex reveals the concentration of that cytokine by comparison with a standard curve.

### 4.7. Statistical Analysis

Comparison of variables within groups was performed using the Student’s *t*-test for dependent samples. Multi-comparison analysis of variables was performed by ANOVA followed by a post hoc multiple comparison test. The level of significance was set at *p*-value < 0.05.

## Figures and Tables

**Table 1:** Characteristics of epithelial ovarian cancer patients included in the study at the time of ascites sampling. Staging classification followed FIGO Committee on Gynecologic Oncology guidelines.

Patient’s Code	Age (Years)	Stage (FIGO)	Histological Classification	Ascites Cytology Results for EOC Cells
21	57	IIB	High-grade serous adenocarcinoma	Negative
24	64	IIIC	Low-grade serous adenocarcinoma	Positive
30	63	IIIC	High-grade serous adenocarcinoma	Negative
40	51	IIIC	High-grade serous adenocarcinoma	Positive
55	56	IIIC	High-grade serous adenocarcinoma	Positive
61	58	IIIC	High-grade serous adenocarcinoma	Negative
70	50	IIIC	Adenocarcinoma NOS	Negative
73	71	IV	High-grade serous adenocarcinoma	Negative
74	43	IIIC	Adenocarcinoma NOS	Positive
75	64	IIIC	High-grade serous adenocarcinoma	Negative
82	45	IIIC	High-grade serous adenocarcinoma	Positive
86	70	IIIC	Adenocarcinoma NOS	Negative
89	79	IV	High-grade serous adenocarcinoma	Positive
93	38	IIIC	Mucinous adenocarcinoma	Negative

NOS = not otherwise specified.

## Supplementary Materials

Supplementary materials can be found at www.mdpi.com/1422-0067/18/5/856/s1.

## Abbreviations

ADCC	Antibody-dependent cellular cytotoxicity
APC-Cy7	Allophycocyanin-cyanine dye
ASC	EOC cell-free ascites
ASC-CA	Ascites of EOC patients with malignant cells
BC	Blood of control women
BP	Blood of patients with EOC
DNAM-1	DNAX accessory molecule-1
EOC	Epithelial ovarian cancer
EpCAM	Epithelial cell adhesion molecule
KIR	Killer Ig-like receptor
LILR-B1	Leukocyte Ig-like receptor B1
vMFI	Variation of mean fluorescence intensity
PE	Phycoerythrin dye
PE-Cy7	Phycoerythrin-cyanine dye
TAL	Tumor-associated lymphocyte
T-reg	Regulatory T-lymphocyte
