# Supplementary material for: Natural Killer Cells Response to IL-2 Stimulation Is Distinct between Ascites with the Presence or Absence of Malignant Cells in Ovarian Cancer Patients
Source: Int J Mol Sci. 2017 May 17;18(5):856. doi: 10.3390/ijms18050856 (PMC5454809; doi:10.3390/ijms18050856)
Supplement: Supplementary file 1 [file ijms-18-00856-s001.pdf]

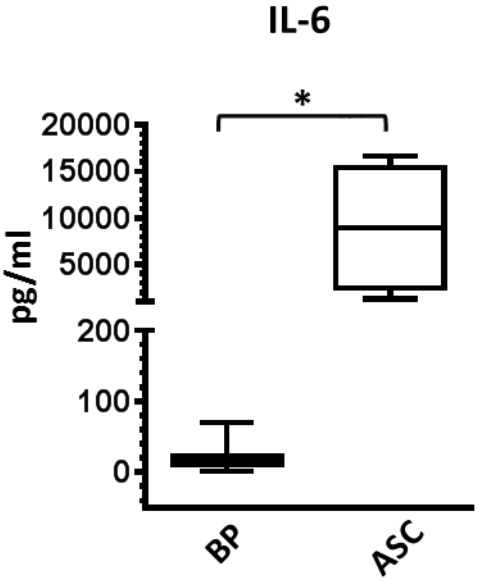

Concentration of IL-6 in the peripheral blood plasma and ascites of EOC patients (n=9). Values are presented in whisker plots as medians. Statistical analyses were performed by t-Student test, and *p* values (* $p<0.05$ on the brackets) indicate significant statistical differences.
